# 4,6-Dimeth­oxy-2-(methyl­sulfon­yl)pyrimidine

**DOI:** 10.1107/S1600536810025067

**Published:** 2010-07-03

**Authors:** Hoong-Kun Fun, Chin Sing Yeap, Sankappa Rai, Arun M Isloor, Prakash Shetty

**Affiliations:** a X-ray Crystallography Unit, School of Physics, Universiti Sains Malaysia, 11800 USM, Penang, Malaysia; b Department of Chemistry, Manipal Institute of Technology, Manipal University, Manipal 576 104, India; c Organic Chemistry Division, Department of Chemistry, National Institute of Technology-Karnataka, Surathkal, Mangalore 575 025, India; d Department of Printing, Manipal Institute of Technology, Manipal University, Manipal 576 104, India

## Abstract

The asymmetric unit of the title compound, C_7_H_10_N_2_O_4_S, comprises of two independent mol­ecules (*A* and *B*) which differ in the orientation of the methyl­sulfonyl group [C—S—C—N = 157.98 (13)° in mol­ecule *A* and 6.09 (18)° in mol­ecule *B*]. In the crystal structure, mol­ecules of type *A* are linked into chains along the *a* axis by inter­molecular C—H⋯O hydrogen bonds. The *B* mol­ecules are linked to these chains by C—H⋯O hydrogen bonds.

## Related literature

For general background and applications of 4,6-dimeth­oxy­pyrimidin-2-yl derivatives, see: Xi *et al.* (2006 ▶); He *et al.* (2007 ▶); Li *et al.* (2006 ▶); Gerorge (1983 ▶).
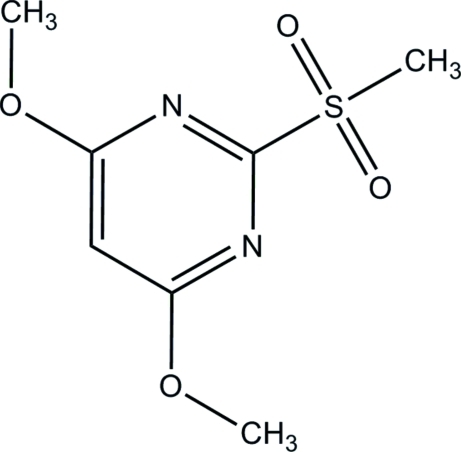


## Experimental

### 

#### Crystal data

C_7_H_10_N_2_O_4_S
*M* *_r_* = 218.23
Triclinic, 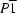

*a* = 8.349 (2) Å
*b* = 11.067 (3) Å
*c* = 11.438 (3) Å
α = 108.457 (8)°
β = 92.774 (8)°
γ = 98.504 (8)°
*V* = 986.4 (4) Å^3^
*Z* = 4
Mo *K*α radiation
μ = 0.32 mm^−1^
*T* = 296 K
0.38 × 0.30 × 0.08 mm

#### Data collection

Bruker APEXII DUO CCD area-detector diffractometer
Absorption correction: multi-scan (*SADABS*; Bruker, 2009 ▶) *T*_min_ = 0.889, *T*_max_ = 0.974
29277 measured reflections
7063 independent reflections
4866 reflections with *I* > 2σ(*I*)
*R*_int_ = 0.042

#### Refinement

*R*[*F* ^2^ > 2σ(*F* ^2^)] = 0.044
*wR*(*F* ^2^) = 0.156
*S* = 1.08
7063 reflections
259 parameters
H-atom parameters constrained
Δρ_max_ = 0.50 e Å^−3^
Δρ_min_ = −0.50 e Å^−3^

### 

Data collection: *APEX2* (Bruker, 2009 ▶); cell refinement: *SAINT* (Bruker, 2009 ▶); data reduction: *SAINT*; program(s) used to solve structure: *SHELXTL* (Sheldrick, 2008 ▶); program(s) used to refine structure: *SHELXTL*; molecular graphics: *SHELXTL*; software used to prepare material for publication: *SHELXTL* and *PLATON* (Spek, 2009 ▶).

## Supplementary Material

Crystal structure: contains datablocks global, I. DOI: 10.1107/S1600536810025067/ci5121sup1.cif

Structure factors: contains datablocks I. DOI: 10.1107/S1600536810025067/ci5121Isup2.hkl

Additional supplementary materials:  crystallographic information; 3D view; checkCIF report

## Figures and Tables

**Table 1:** Hydrogen-bond geometry (Å, °)

*D*—H⋯*A*	*D*—H	H⋯*A*	*D*⋯*A*	*D*—H⋯*A*
C3*A*—H3*AA*⋯O2*A*^i^	0.93	2.42	3.336 (2)	169
C5*A*—H5*AC*⋯O2*B*^ii^	0.96	2.55	3.303 (3)	135
C7*A*—H7*AA*⋯O4*A*^iii^	0.96	2.50	3.426 (2)	161

Symmetry codes: (i) 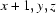; (ii) 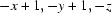; (iii) 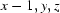.

**Fig. 1.**
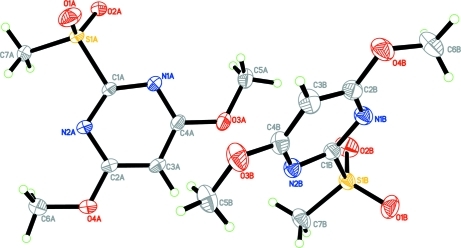
The two independent molecules of the title compound with atom labels and 30% probability displacement ellipsoids for non-H atoms.

**Fig. 2.**
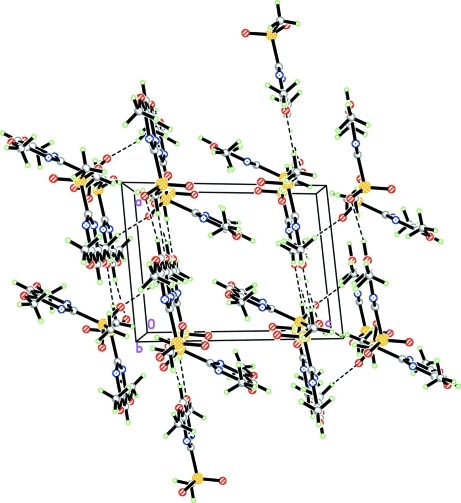
The crystal packing of title compound, viewed down the b axis showing chains along the a axis.

## supplementary crystallographic information

### Comment

Compounds containing 4,6-dimethoxypyrimidin-2-yl moieties display excellent herbicidal activity (Xi *et al.*, 2006). Most sulfonylurea herbicides and all pyrimidinylbenzoate herbicides (He *et al.*, 2007), such as nicofulfuron, amidosulfuron, halopyrazosulfuron, ethoxysulfuron, pyriminobac-methyl and pyriftalid, possess 4,6-dimethoxypyrimidin-2-yl groups (Li *et al.*, 2006), while sulfometuron-methyl, a kind of sulfonylurea, contains a 4,6-dimethylpyrimidin-2-yl group, which suggests that the two disubstituted pyrimidin- 2-yl groups possess high biological activity (Gerorge, 1983).

There are two molecules, *A* and *B*, in the asymmetric unit (Fig. 1) of the title compound. The molecules A and B differ in the orientation of the methylsulfonyl group [C7A—S1A—C1A—N1A = 157.98 (13)° and C7B—S1B—C1B—N2B = 6.09 (18)°]

In the crystal structure, the *A* molecules are linked into chains along *a* axis by intermolecular C3A—H3AA···O2A and C7A—H7AA···O4A hydrogen bonds. The *B* molecules are linked to these chains by intermolecular C5A—H5AC···O2B hydrogen bonds.

### Experimental

Periodic acid (2.63 mmol, 600 mg) was dissolved in acetonitrile (6 ml) by stirring at room temperature for 1 h. To this solution, chromium trioxide (0.125 mmol, 12.5 mg) was added and stirred for 5 min to give a clear orange solution. H_5_IO_6_/CrO_3_ solution (1.7 ml) was added to a solution of 4,6-dimethoxy-2-methylmercaptopyrimidine (0.23 mmol) in ethyl acetate and was stirred at room temperature for 30 min. The reaction mixture was quenched with saturated sodium sulphite and was loaded on to a silica column. The column was eluted with acetone to obtain 4,6-dimethoxy-2-methylsulfonylpyrimidine. Single crystals were recrystallized from an dichloromethane solution (yield: 87%, m.p. 402–405 K).

### Refinement

All H atoms were positioned geometrically [C–H = 0.93 or 0.96 Å] and refined using a riding model, with *U*_iso_(H) = 1.2 or 1.5*U*_eq_(C). A rotating-group model was applied for the methyl groups.

### Crystal data

**Table:**

C_7_H_10_N_2_O_4_S	*Z* = 4
*M**_r_* = 218.23	*F*(000) = 456
Triclinic, *P*1	*D*_x_ = 1.470 Mg m^−^^3^
Hall symbol: -P 1	Mo *K*α radiation, λ = 0.71073 Å
*a* = 8.349 (2) Å	Cell parameters from 8362 reflections
*b* = 11.067 (3) Å	θ = 2.2–32.1°
*c* = 11.438 (3) Å	µ = 0.32 mm^−^^1^
α = 108.457 (8)°	*T* = 296 K
β = 92.774 (8)°	Plate, colourless
γ = 98.504 (8)°	0.38 × 0.30 × 0.08 mm
*V* = 986.4 (4) Å^3^	

### Data collection

**Table:**

Bruker APEXII DUO CCD area-detector diffractometer	7063 independent reflections
Radiation source: fine-focus sealed tube	4866 reflections with *I* > 2σ(*I*)
graphite	*R*_int_ = 0.042
φ and ω scans	θ_max_ = 32.5°, θ_min_ = 1.9°
Absorption correction: multi-scan (*SADABS*; Bruker, 2009)	*h* = −12→12
*T*_min_ = 0.889, *T*_max_ = 0.974	*k* = −16→16
29277 measured reflections	*l* = −17→16

### Refinement

**Table:**

Refinement on *F*^2^	Primary atom site location: structure-invariant direct methods
Least-squares matrix: full	Secondary atom site location: difference Fourier map
*R*[*F*^2^ > 2σ(*F*^2^)] = 0.044	Hydrogen site location: inferred from neighbouring sites
*wR*(*F*^2^) = 0.156	H-atom parameters constrained
*S* = 1.08	*w* = 1/[σ^2^(*F*_o_^2^) + (0.0771*P*)^2^ + 0.1962*P*] where *P* = (*F*_o_^2^ + 2*F*_c_^2^)/3
7063 reflections	(Δ/σ)_max_ = 0.001
259 parameters	Δρ_max_ = 0.50 e Å^−^^3^
0 restraints	Δρ_min_ = −0.50 e Å^−^^3^

### Special details

**Table:**

Geometry. All e.s.d.'s (except the e.s.d. in the dihedral angle between two l.s. planes) are estimated using the full covariance matrix. The cell e.s.d.'s are taken into account individually in the estimation of e.s.d.'s in distances, angles and torsion angles; correlations between e.s.d.'s in cell parameters are only used when they are defined by crystal symmetry. An approximate (isotropic) treatment of cell e.s.d.'s is used for estimating e.s.d.'s involving l.s. planes.
Refinement. Refinement of *F*^2^ against ALL reflections. The weighted *R*-factor *wR* and goodness of fit *S* are based on *F*^2^, conventional *R*-factors *R* are based on *F*, with *F* set to zero for negative *F*^2^. The threshold expression of *F*^2^ > σ(*F*^2^) is used only for calculating *R*-factors(gt) *etc*. and is not relevant to the choice of reflections for refinement. *R*-factors based on *F*^2^ are statistically about twice as large as those based on *F*, and *R*- factors based on ALL data will be even larger.

### Fractional atomic coordinates and isotropic or equivalent isotropic displacement parameters (Å^2^)

**Table:**

	*x*	*y*	*z*	*U*_iso_*/*U*_eq_	
S1A	−0.01034 (4)	0.04422 (4)	0.19330 (4)	0.03856 (12)	
O1A	−0.02361 (19)	0.10070 (16)	0.32304 (15)	0.0632 (4)	
O2A	−0.08995 (15)	0.09457 (13)	0.10934 (15)	0.0518 (3)	
O3A	0.50597 (16)	0.29863 (13)	0.14202 (15)	0.0528 (3)	
O4A	0.51835 (16)	−0.11116 (13)	0.16945 (16)	0.0543 (3)	
N1A	0.26662 (16)	0.17382 (13)	0.16027 (13)	0.0362 (3)	
N2A	0.27464 (15)	−0.03547 (13)	0.17789 (13)	0.0364 (3)	
C1A	0.20400 (17)	0.06224 (14)	0.17287 (14)	0.0334 (3)	
C2A	0.43408 (19)	−0.01755 (16)	0.16718 (16)	0.0386 (3)	
C3A	0.51867 (19)	0.09495 (18)	0.15559 (17)	0.0435 (4)	
H3AA	0.6304	0.1068	0.1502	0.052*	
C4A	0.42742 (19)	0.18858 (16)	0.15246 (15)	0.0378 (3)	
C5A	0.4131 (3)	0.3951 (2)	0.1326 (3)	0.0608 (5)	
H5AA	0.4838	0.4653	0.1194	0.091*	
H5AB	0.3635	0.4268	0.2078	0.091*	
H5AC	0.3300	0.3577	0.0642	0.091*	
C6A	0.4289 (3)	−0.2300 (2)	0.1768 (3)	0.0630 (6)	
H6AA	0.5032	−0.2872	0.1814	0.095*	
H6AB	0.3525	−0.2704	0.1045	0.095*	
H6AC	0.3714	−0.2116	0.2494	0.095*	
C7A	−0.0758 (2)	−0.12239 (17)	0.1485 (2)	0.0476 (4)	
H7AA	−0.1909	−0.1400	0.1536	0.071*	
H7AB	−0.0197	−0.1571	0.2024	0.071*	
H7AC	−0.0529	−0.1618	0.0648	0.071*	
S1B	0.91824 (6)	0.54889 (4)	0.18573 (4)	0.04122 (12)	
O1B	1.0732 (2)	0.63051 (15)	0.21079 (16)	0.0655 (4)	
O2B	0.8041 (2)	0.56321 (17)	0.09628 (13)	0.0639 (4)	
O3B	0.7212 (2)	0.42513 (15)	0.54172 (14)	0.0673 (5)	
O4B	0.6688 (2)	0.83263 (14)	0.52687 (15)	0.0629 (4)	
N1B	0.78624 (19)	0.69578 (14)	0.36974 (14)	0.0437 (3)	
N2B	0.81160 (19)	0.48548 (14)	0.37722 (13)	0.0414 (3)	
C1B	0.8263 (2)	0.57971 (16)	0.32927 (15)	0.0382 (3)	
C2B	0.7173 (2)	0.71868 (18)	0.47615 (17)	0.0471 (4)	
C3B	0.6896 (3)	0.6287 (2)	0.53590 (18)	0.0574 (5)	
H3BA	0.6383	0.6454	0.6084	0.069*	
C4B	0.7415 (3)	0.51237 (18)	0.48331 (17)	0.0479 (4)	
C5B	0.7711 (4)	0.3023 (2)	0.4853 (2)	0.0658 (6)	
H5BA	0.7710	0.2564	0.5438	0.099*	
H5BB	0.8787	0.3163	0.4607	0.099*	
H5BC	0.6967	0.2525	0.4138	0.099*	
C6B	0.7263 (4)	0.9364 (2)	0.4804 (2)	0.0717 (7)	
H6BA	0.7075	1.0168	0.5370	0.108*	
H6BB	0.6689	0.9210	0.4009	0.108*	
H6BC	0.8408	0.9406	0.4725	0.108*	
C7B	0.9425 (3)	0.38667 (19)	0.1441 (2)	0.0596 (6)	
H7BA	0.9860	0.3620	0.0655	0.089*	
H7BB	0.8389	0.3332	0.1384	0.089*	
H7BC	1.0160	0.3758	0.2058	0.089*	

### Atomic displacement parameters (Å^2^)

**Table:**

	*U*^11^	*U*^22^	*U*^33^	*U*^12^	*U*^13^	*U*^23^
S1A	0.02471 (18)	0.03529 (19)	0.0583 (3)	0.00757 (13)	0.01261 (15)	0.01667 (17)
O1A	0.0498 (8)	0.0687 (9)	0.0636 (9)	0.0117 (7)	0.0238 (7)	0.0079 (7)
O2A	0.0291 (6)	0.0482 (7)	0.0888 (10)	0.0116 (5)	0.0074 (6)	0.0351 (7)
O3A	0.0338 (6)	0.0503 (7)	0.0794 (10)	−0.0034 (5)	0.0040 (6)	0.0332 (7)
O4A	0.0313 (6)	0.0516 (7)	0.0890 (10)	0.0153 (5)	0.0114 (6)	0.0311 (7)
N1A	0.0264 (6)	0.0368 (6)	0.0450 (7)	0.0026 (5)	0.0036 (5)	0.0143 (5)
N2A	0.0255 (6)	0.0385 (6)	0.0472 (7)	0.0077 (5)	0.0063 (5)	0.0156 (6)
C1A	0.0226 (6)	0.0364 (7)	0.0410 (8)	0.0041 (5)	0.0052 (5)	0.0126 (6)
C2A	0.0265 (7)	0.0440 (8)	0.0473 (9)	0.0093 (6)	0.0055 (6)	0.0159 (7)
C3A	0.0235 (7)	0.0518 (9)	0.0575 (10)	0.0048 (6)	0.0060 (6)	0.0216 (8)
C4A	0.0283 (7)	0.0419 (8)	0.0433 (8)	0.0003 (6)	0.0034 (6)	0.0167 (6)
C5A	0.0500 (11)	0.0536 (11)	0.0890 (16)	0.0007 (9)	0.0077 (10)	0.0411 (11)
C6A	0.0476 (11)	0.0511 (11)	0.1001 (18)	0.0169 (9)	0.0138 (11)	0.0339 (11)
C7A	0.0319 (8)	0.0385 (8)	0.0761 (13)	0.0027 (6)	0.0102 (8)	0.0249 (8)
S1B	0.0485 (2)	0.0379 (2)	0.0431 (2)	0.01011 (16)	0.01501 (17)	0.01859 (16)
O1B	0.0583 (9)	0.0610 (9)	0.0768 (10)	−0.0036 (7)	0.0250 (8)	0.0258 (8)
O2B	0.0830 (12)	0.0751 (10)	0.0444 (7)	0.0300 (9)	0.0083 (7)	0.0269 (7)
O3B	0.1028 (14)	0.0552 (8)	0.0543 (8)	0.0157 (8)	0.0304 (8)	0.0283 (7)
O4B	0.0800 (11)	0.0481 (8)	0.0605 (9)	0.0220 (7)	0.0250 (8)	0.0102 (7)
N1B	0.0480 (8)	0.0403 (7)	0.0440 (8)	0.0111 (6)	0.0096 (6)	0.0132 (6)
N2B	0.0465 (8)	0.0401 (7)	0.0396 (7)	0.0060 (6)	0.0089 (6)	0.0160 (6)
C1B	0.0382 (8)	0.0395 (8)	0.0376 (8)	0.0067 (6)	0.0064 (6)	0.0134 (6)
C2B	0.0506 (10)	0.0433 (9)	0.0442 (9)	0.0100 (7)	0.0103 (7)	0.0084 (7)
C3B	0.0775 (15)	0.0518 (11)	0.0430 (10)	0.0119 (10)	0.0243 (9)	0.0126 (8)
C4B	0.0593 (11)	0.0450 (9)	0.0394 (9)	0.0040 (8)	0.0115 (8)	0.0151 (7)
C5B	0.0955 (19)	0.0489 (11)	0.0579 (13)	0.0094 (11)	0.0111 (12)	0.0255 (10)
C6B	0.104 (2)	0.0477 (11)	0.0687 (14)	0.0285 (12)	0.0197 (13)	0.0182 (10)
C7B	0.0803 (15)	0.0423 (10)	0.0653 (13)	0.0231 (9)	0.0347 (11)	0.0206 (9)

### Geometric parameters (Å, °)

**Table:**

S1A—O1A	1.4334 (16)	S1B—O1B	1.4234 (16)
S1A—O2A	1.4356 (14)	S1B—O2B	1.4260 (16)
S1A—C7A	1.7429 (18)	S1B—C7B	1.751 (2)
S1A—C1A	1.8059 (15)	S1B—C1B	1.8018 (17)
O3A—C4A	1.338 (2)	O3B—C4B	1.333 (2)
O3A—C5A	1.436 (3)	O3B—C5B	1.441 (3)
O4A—C2A	1.342 (2)	O4B—C2B	1.342 (2)
O4A—C6A	1.443 (2)	O4B—C6B	1.442 (3)
N1A—C1A	1.322 (2)	N1B—C1B	1.320 (2)
N1A—C4A	1.339 (2)	N1B—C2B	1.339 (2)
N2A—C1A	1.321 (2)	N2B—C1B	1.317 (2)
N2A—C2A	1.334 (2)	N2B—C4B	1.340 (2)
C2A—C3A	1.386 (2)	C2B—C3B	1.375 (3)
C3A—C4A	1.382 (2)	C3B—C4B	1.381 (3)
C3A—H3AA	0.93	C3B—H3BA	0.93
C5A—H5AA	0.96	C5B—H5BA	0.96
C5A—H5AB	0.96	C5B—H5BB	0.96
C5A—H5AC	0.96	C5B—H5BC	0.96
C6A—H6AA	0.96	C6B—H6BA	0.96
C6A—H6AB	0.96	C6B—H6BB	0.96
C6A—H6AC	0.96	C6B—H6BC	0.96
C7A—H7AA	0.96	C7B—H7BA	0.96
C7A—H7AB	0.96	C7B—H7BB	0.96
C7A—H7AC	0.96	C7B—H7BC	0.96
			
O1A—S1A—O2A	117.87 (10)	O1B—S1B—O2B	117.30 (11)
O1A—S1A—C7A	109.37 (10)	O1B—S1B—C7B	110.02 (12)
O2A—S1A—C7A	109.13 (9)	O2B—S1B—C7B	109.13 (12)
O1A—S1A—C1A	107.07 (8)	O1B—S1B—C1B	107.47 (9)
O2A—S1A—C1A	108.07 (8)	O2B—S1B—C1B	107.25 (9)
C7A—S1A—C1A	104.49 (8)	C7B—S1B—C1B	104.92 (9)
C4A—O3A—C5A	118.66 (14)	C4B—O3B—C5B	118.20 (16)
C2A—O4A—C6A	117.66 (14)	C2B—O4B—C6B	117.81 (17)
C1A—N1A—C4A	113.74 (14)	C1B—N1B—C2B	113.47 (15)
C1A—N2A—C2A	113.88 (14)	C1B—N2B—C4B	113.85 (15)
N2A—C1A—N1A	130.25 (14)	N2B—C1B—N1B	130.63 (16)
N2A—C1A—S1A	115.13 (11)	N2B—C1B—S1B	115.98 (12)
N1A—C1A—S1A	114.55 (11)	N1B—C1B—S1B	113.38 (13)
N2A—C2A—O4A	119.16 (15)	N1B—C2B—O4B	119.64 (18)
N2A—C2A—C3A	123.00 (15)	N1B—C2B—C3B	122.83 (17)
O4A—C2A—C3A	117.83 (15)	O4B—C2B—C3B	117.51 (17)
C4A—C3A—C2A	116.11 (15)	C2B—C3B—C4B	116.88 (17)
C4A—C3A—H3AA	121.9	C2B—C3B—H3BA	121.6
C2A—C3A—H3AA	121.9	C4B—C3B—H3BA	121.6
O3A—C4A—N1A	119.54 (15)	O3B—C4B—N2B	119.62 (17)
O3A—C4A—C3A	117.47 (15)	O3B—C4B—C3B	118.09 (17)
N1A—C4A—C3A	122.99 (15)	N2B—C4B—C3B	122.29 (17)
O3A—C5A—H5AA	109.5	O3B—C5B—H5BA	109.5
O3A—C5A—H5AB	109.5	O3B—C5B—H5BB	109.5
H5AA—C5A—H5AB	109.5	H5BA—C5B—H5BB	109.5
O3A—C5A—H5AC	109.5	O3B—C5B—H5BC	109.5
H5AA—C5A—H5AC	109.5	H5BA—C5B—H5BC	109.5
H5AB—C5A—H5AC	109.5	H5BB—C5B—H5BC	109.5
O4A—C6A—H6AA	109.5	O4B—C6B—H6BA	109.5
O4A—C6A—H6AB	109.5	O4B—C6B—H6BB	109.5
H6AA—C6A—H6AB	109.5	H6BA—C6B—H6BB	109.5
O4A—C6A—H6AC	109.5	O4B—C6B—H6BC	109.5
H6AA—C6A—H6AC	109.5	H6BA—C6B—H6BC	109.5
H6AB—C6A—H6AC	109.5	H6BB—C6B—H6BC	109.5
S1A—C7A—H7AA	109.5	S1B—C7B—H7BA	109.5
S1A—C7A—H7AB	109.5	S1B—C7B—H7BB	109.5
H7AA—C7A—H7AB	109.5	H7BA—C7B—H7BB	109.5
S1A—C7A—H7AC	109.5	S1B—C7B—H7BC	109.5
H7AA—C7A—H7AC	109.5	H7BA—C7B—H7BC	109.5
H7AB—C7A—H7AC	109.5	H7BB—C7B—H7BC	109.5
			
C2A—N2A—C1A—N1A	−0.7 (3)	C4B—N2B—C1B—N1B	1.4 (3)
C2A—N2A—C1A—S1A	−177.62 (12)	C4B—N2B—C1B—S1B	−179.50 (13)
C4A—N1A—C1A—N2A	−0.6 (3)	C2B—N1B—C1B—N2B	−1.2 (3)
C4A—N1A—C1A—S1A	176.33 (12)	C2B—N1B—C1B—S1B	179.75 (13)
O1A—S1A—C1A—N2A	91.31 (14)	O1B—S1B—C1B—N2B	−110.99 (15)
O2A—S1A—C1A—N2A	−140.77 (13)	O2B—S1B—C1B—N2B	122.06 (15)
C7A—S1A—C1A—N2A	−24.64 (15)	C7B—S1B—C1B—N2B	6.09 (18)
O1A—S1A—C1A—N1A	−86.07 (14)	O1B—S1B—C1B—N1B	68.23 (16)
O2A—S1A—C1A—N1A	41.86 (14)	O2B—S1B—C1B—N1B	−58.72 (16)
C7A—S1A—C1A—N1A	157.98 (13)	C7B—S1B—C1B—N1B	−174.69 (15)
C1A—N2A—C2A—O4A	−179.19 (16)	C1B—N1B—C2B—O4B	−179.21 (18)
C1A—N2A—C2A—C3A	1.8 (2)	C1B—N1B—C2B—C3B	−0.8 (3)
C6A—O4A—C2A—N2A	3.3 (3)	C6B—O4B—C2B—N1B	−14.0 (3)
C6A—O4A—C2A—C3A	−177.70 (18)	C6B—O4B—C2B—C3B	167.5 (2)
N2A—C2A—C3A—C4A	−1.6 (3)	N1B—C2B—C3B—C4B	2.1 (3)
O4A—C2A—C3A—C4A	179.42 (16)	O4B—C2B—C3B—C4B	−179.4 (2)
C5A—O3A—C4A—N1A	−3.4 (3)	C5B—O3B—C4B—N2B	−2.2 (3)
C5A—O3A—C4A—C3A	177.06 (19)	C5B—O3B—C4B—C3B	178.3 (2)
C1A—N1A—C4A—O3A	−178.65 (15)	C1B—N2B—C4B—O3B	−179.31 (19)
C1A—N1A—C4A—C3A	0.8 (2)	C1B—N2B—C4B—C3B	0.2 (3)
C2A—C3A—C4A—O3A	179.63 (16)	C2B—C3B—C4B—O3B	177.7 (2)
C2A—C3A—C4A—N1A	0.1 (3)	C2B—C3B—C4B—N2B	−1.8 (3)

### Hydrogen-bond geometry (Å, °)

**Table:**

*D*—H···*A*	*D*—H	H···*A*	*D*···*A*	*D*—H···*A*
C3A—H3AA···O2A^i^	0.93	2.42	3.336 (2)	169
C5A—H5AC···O2B^ii^	0.96	2.55	3.303 (3)	135
C7A—H7AA···O4A^iii^	0.96	2.50	3.426 (2)	161

Symmetry codes: (i) *x*+1, *y*, *z*; (ii) −*x*+1, −*y*+1, −*z*; (iii) *x*−1, *y*, *z*.
